# Supplementary material for: The subset of peroxisomal tail-anchored proteins do not reach peroxisomes via ER, instead mitochondria can be involved
Source: PLoS One. 2023 Dec 1;18(12):e0295047. doi: 10.1371/journal.pone.0295047 (PMC10691693; doi:10.1371/journal.pone.0295047)
Supplement: S1 Raw images — (PDF) [file pone.0295047.s002.pdf]

Anti-PEX3 antibody

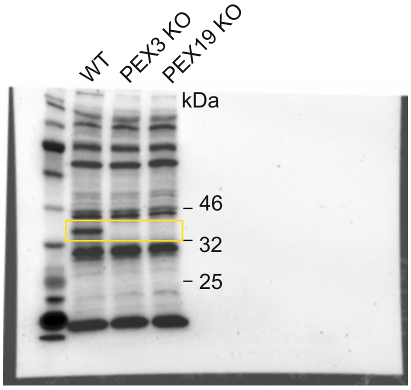

Anti-PEX3 antibody
(overexposed image)

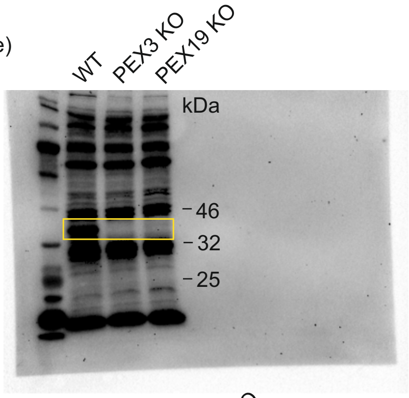

Anti-PEX19 antibody

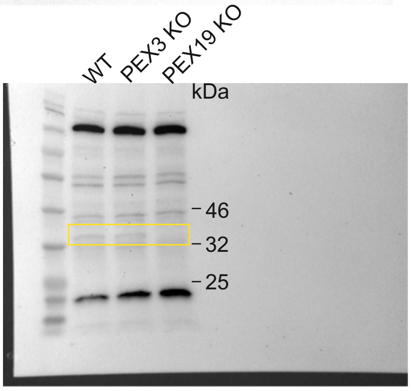

Anti-actin antibody

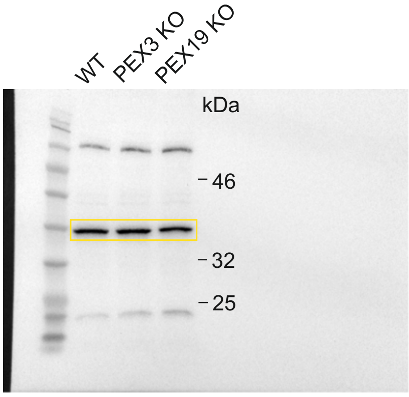

Fig S1. Uncropped blot images underlying Fig 1A in the main text.
Immunoblotting analysis of HEK293T WT, PEX3 KO or PEX19 KO whole cell lysate using anti-PEX3 antibody (Schmidt et al., 2012), anti-PEX19 antibody (Abcam, 137072) or anti-actin antibody (Cell Signaling Technology, 3700). In every image, the first lane represents the protein ladder (Color Prestained Protein Standard, Broad Range (11–245 kDa), NEB#7712). Portions on the gels enclosed in the yellow square were used for the figure generation.

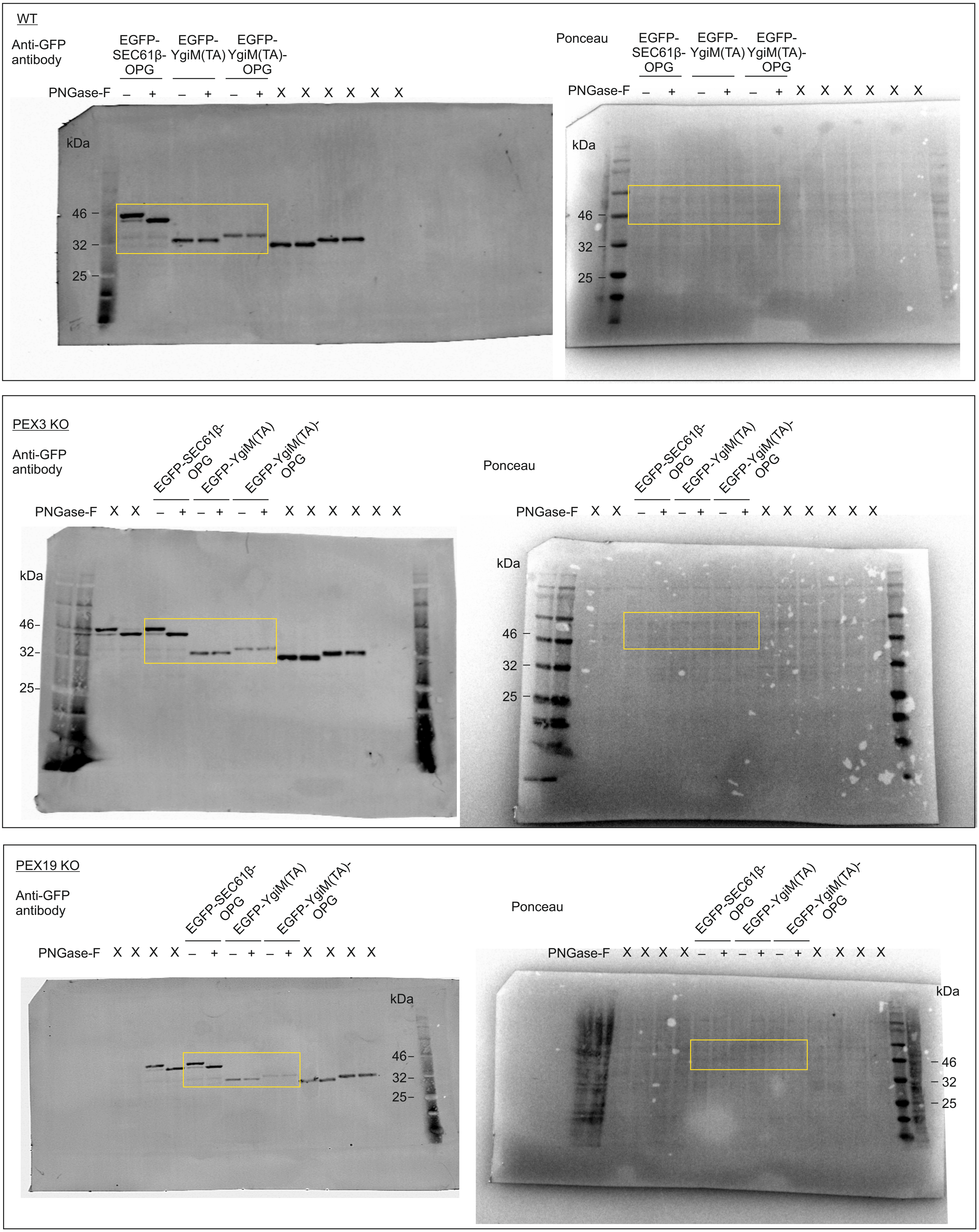

Fig S2. Uncropped and unadjusted blot images, underlying Fig 1E in the main text.
Glycosylation analysis in HEK293T WT, PEX3 KO or PEX19 KO cells by immunoblotting using anti-GFP antibody (Cell Signaling Technology, 2956). Ponceau S staining of the membranes was used as loading control. Portions on the gels enclosed in the yellow square were used for the figure generation.

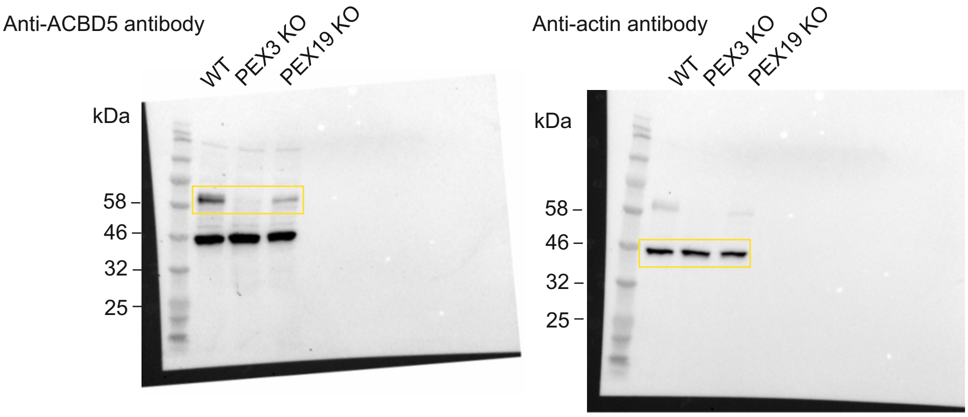

Fig S3. Uncropped and unadjusted blot images, underlying Fig 3E in the main text.
ACBD5 protein levels in HEK293T WT, PEX3 KO, PEX19 KO cells (Atlas Antibodies, HPA012145). Anti-actin antibody was used to detect actin (Cell Signaling Technology, 3700). Portions on the gels enclosed in the yellow square were used for the figure generation.

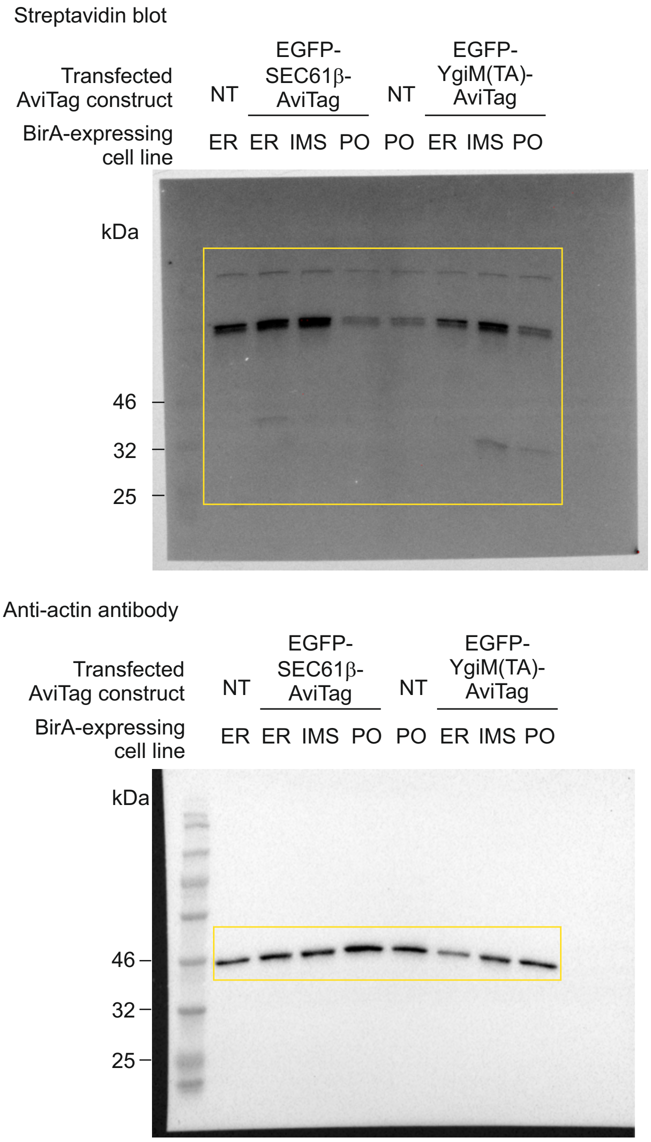

Fig S4. Uncropped and unadjusted blot images, underlying Fig 4E in the main text.
Cell lysates were analyzed by immunoblotting using streptavidin (LI-COR, 926-32230) with IR imaging on the iBright Imaging Systems. Anti-actin antibody was used to detect actin (Cell Signaling Technology, 3700). Portions on the gels enclosed in the yellow square were used for the figure generation.
