# Supplementary material for: The subset of peroxisomal tail-anchored proteins do not reach peroxisomes via ER, instead mitochondria can be involved
Source: PLoS One. 2023 Dec 1;18(12):e0295047. doi: 10.1371/journal.pone.0295047 (PMC10691693; doi:10.1371/journal.pone.0295047)
Supplement: Attachment — Submitted filename: Response to reviewers.docx [file pone.0295047.s003.docx]

The authors would like to thank the Reviewers for the insightful comments and suggestions, which have helped to improve the manuscript.

Reviewers' comments: 
 
Reviewer's Responses to Questions

**Comments to the Author** 
 
1. Is the manuscript technically sound, and do the data support the conclusions? 
 
The manuscript must describe a technically sound piece of scientific research with data that supports the conclusions. Experiments must have been conducted rigorously, with appropriate controls, replication, and sample sizes. The conclusions must be drawn appropriately based on the data presented.

Reviewer #1: Partly

Reviewer #2: Yes

2. Has the statistical analysis been performed appropriately and rigorously?

Reviewer #1: Yes

Reviewer #2: Yes

3. Have the authors made all data underlying the findings in their manuscript fully available? 
 
The [PLOS Data policy](http://www.plosone.org/static/policies.action#sharing) requires authors to make all data underlying the findings described in their manuscript fully available without restriction, with rare exception (please refer to the Data Availability Statement in the manuscript PDF file). The data should be provided as part of the manuscript or its supporting information, or deposited to a public repository. For example, in addition to summary statistics, the data points behind means, medians and variance measures should be available. If there are restrictions on publicly sharing data—e.g. participant privacy or use of data from a third party—those must be specified.

Reviewer #1: Yes

Reviewer #2: Yes

4. Is the manuscript presented in an intelligible fashion and written in standard English? 
 
PLOS ONE does not copyedit accepted manuscripts, so the language in submitted articles must be clear, correct, and unambiguous. Any typographical or grammatical errors should be corrected at revision, so please note any specific errors here.

Reviewer #1: Yes

Reviewer #2: Yes

5. Review Comments to the Author 
 
Please use the space provided to explain your answers to the questions above. You may also include additional comments for the author, including concerns about dual publication, research ethics, or publication ethics. (Please upload your review as an attachment if it exceeds 20,000 characters)

Reviewer #1: In this manuscript, Somborac et al. reported a study on the delivery pathway of the tail-anchored (TA) proteins to peroxisomes in mammalian cells. Peroxisome biogenesis is an important research area since its dysfunction can be a reason for both rare and prevalent diseases. From that point of view, the research presented in this manuscript on how peroxisomal membrane proteins (PMPs) are targeted to the membrane of peroxisome would be a nice addition to the understanding of peroxisome biogenesis. However, some sections in the manuscript are confusing and require further explanation. Below are the comments: 
 
1. An important observation of this manuscript is that the peroxisomal TA proteins may be targeted to the mitochondria but not to the ER before their final destination to the peroxisome. However, the authors never mentioned the outcomes or shortcomings (if any) if the TA proteins are transported to the ER prior to their delivery to peroxisomes. How do mitochondria play a beneficial (if any) role in being a targeted organelle for TA protein before its final delivery to the peroxisomal membrane?

We appreciate highlighting this important observation. The targeting of TA proteins to specific organelles is a complex process that is essential for maintaining cellular homeostasis. TA proteins can be trafficked to various organelles, including the endoplasmic reticulum (ER), mitochondria, and peroxisomes (PMID: 17629691).

In yeast, the GET pathway mediates the targeting and insertion of TA proteins into the ER membrane, highlighting the ER's ability to process TA proteins (PMID: 17382883). In mammalian cells, the mechanisms are not fully understood. While some TA proteins may be targeted directly to their final destination, others transit through intermediary organelles such as the ER or mitochondria (PMID: [28108524](https://pubmed.ncbi.nlm.nih.gov/28108524);, PMID: [25177298](https://pubmed.ncbi.nlm.nih.gov/25177298), PMID: 28146471).

The potential advantages of TA protein transit through the mitochondria prior to peroxisome localization are numerous. Mitochondria and peroxisomes share several functional overlaps, including fatty acid and reactive oxygen species (ROS) metabolism (PMID: 25687155, PMID: 25307522). During mitochondrial transit, TA proteins may undergo specific post-translational modifications or protein-protein interactions that prime them for their peroxisomal function. Additionally, this transit could serve as a quality control step, ensuring that only correctly folded and functional TA proteins reach the peroxisome.

The question of why some TA proteins bypass the ER *en route* to the peroxisome remains elusive. Given the ER's well-established role in protein processing (PMID: 11269317, PMID: [23396477](https://pubmed.ncbi.nlm.nih.gov/23396477), PMID: [28187289](https://pubmed.ncbi.nlm.nih.gov/28187289)), it would be reasonable to expect that the ER serves as a ubiquitous transit point for TA proteins. However, as our data suggests, some TA proteins, such as ACBD5, are targeted to the mitochondria before their final destination in the peroxisome and appear to bypass the ER altogether. This process could be attributed to the specific requirements of these proteins or the cells in which they function. For example, direct targeting to the mitochondria may expedite the delivery of these proteins, ensuring timely responses to cellular cues. In addition, bypassing the ER may prevent potential mistargeting or delays associated with the ER's extensive protein processing machinery.

Now we added some of these hypotheses to the Discussion part.

2. In figure 1b, authors need to describe the microscope image in detail. What are these cyan and magenta-colored objects mean? The same comment goes for figure 1d. For general readers, authors need to explain the data and point out the observation(s) either in the figure legend or in the results section.

We thank Reviewer for this comment. Now we have provided more description of the figures explaining the data and observation.

3. In the case of figure 1e, a loading control is necessary. The level of TA proteins is different in different cell lines. Moreover, this glycosylation assay requires proper insertion of the membrane proteins where the glycosylation tag is exposed to the ER lumen. How do the authors confirm that in the absence of peroxisomes, the TA proteins are not aggregated, which of course would fail the glycosylation assay?

We appreciate Reviewer for the comment. Now we have added Ponceau S staining as a loading control for each presented blot in Fig 1E. The loading seems to be equal between different samples. We do see that the constructs are expressed at a different level. However, in this assay we did not compare the expression levels, rather we aimed to detect the glycosylation by a western blot band shift.

Indeed, the proper insertion of the construct into ER is required for successful glycosylation. Using glycosylation assay, we aimed to investigate whether YgiM(TA) targets ER. We showed that EGFP-YgiM(TA)-OPG is not glycosylated in HEK293T cells, suggesting that EGFP-YgiM(TA)-OPG does not target ER at all, or its insertion is not compatible with the glycosylation.

Therefore, next, we used alternative methods such as confocal immunofluorescence, immunoelectron microscopy or proximity biotinylation assays to identify the localization of the EGFP-YgiM(TA).

Importantly, using electron microscopy we did not observe aggregates of EGFP-YgiM(TA) in PEX3 KO or PEX19 KO cells (Fig 2A, 2B), indicating that EGFP-YgiM(TA) is not aggregated in the absence of peroxisomes. In the glycosylation assay we used EGFP-YgiM(TA) fused to OPG. This construct differs from the construct that we used for electron microscopy by the presence of C-terminal OPG. However, OPG is a relatively small tag (13 amino acids) that was shown to not affect targeting of TA proteins (PMID: 21466504, PMID: 20516149).

4. In figure 2b, the colocalization of YgiM(TA) with both ER and mitochondria contradicts the result of the glycosylation assay that was presented in figure 1e. If PEX3 KO or PEX19 KO cells are showing colocalization of YgiM(TA) with ER, why the glycosylation assay showed no glycosylation bands for YgiM(TA)-OPG protein in those two cell lines? Authors need to justify this observation.

We appreciate Reviewer for this comment. Indeed, our confocal microscopy images show colocalization of YgiM(TA) with both ER and mitochondria in PEX3 KO or PEX19 KO cells (Fig 2C). On the other hand, we did not observe glycosylation of YgiM(TA)-OPG (Fig 1E), suggesting that YgiM(TA)-OPG does not transit through the ER. This seemed contradiction can be addressed by considering the limitations of the two techniques we used. Confocal microscopy allows us to visualize the spatial distribution of proteins within the cell, while powerful, it has resolution limitations that might affect the interpretation of colocalization studies (PMID: 21209361). The resolution limit also prevents us from claiming that the YgiM(TA) is inserted in the ER, instead of just near it. Thus, the observed colocalization might be due to close proximity or interactions between the organelles, rather than a direct insertion of the YgiM(TA) into the ER. Additionally, we quantified the colocalization between the ER and mitochondria. The results showed that around 50 % of the calnexin is colocalized with ATPIF1 (Author response Figure 1). This explains why we observed the colocalization between the ER and YgiM(TA) (Fig 2B).

Importantly, the coefficient of calnexin / ATPIF1 colocalization was not affected by PEX3 KO or PEX19 KO, suggesting that the significant increase in colocalization between YgiM(TA) and ATPIF1 was not due to the changes in the ER / mitochondria interactions.

**Author response Figure 1**

**Author response Figure 1. Colocalization between ER and mitochondria in WT, PEX3 KO or PEX19 KO HEK293T cells.**

Colocalization of ER (visualized by anti-calnexin Ab) with mitochondria (visualized by anti-ATPIF1) was analyzed by Mander's overlap coefficient using confocal microscopy images. For each sample 5-6 fields of view were analyzed, each view captured approximately 50 cells. The data are presented as mean ± SD, ns, not significant as compared to WT cells (unpaired t-tests, n = 5-6) as compared to WT cells (unpaired t-tests, n = 5-6).

On the other hand, in the glycosylation assay, the absence of glycosylation bands in the assay indicates that the protein did not undergo glycosylation in the ER. This is a more direct measure of whether the protein transits through the ER. However, we do acknowledge that the absence of glycosylation does not necessarily mean the absence of insertion, therefore we aimed to address this issue with alternative methods, such as high-resolution electron microscopy and proximity labeling.

Importantly, although confocal microscopy showed the colocalization between YgiM(TA) and ER in PEX3 KO or PEX19 KO cells, quantitatively most of YgiM(TA) is localized to mitochondria in these cells. In addition, the immuno-electron microscopy clearly shows the presence of YgiM(TA) on mitochondrial membranes, and not in ER (Fig 2D, 2E).

Overall, each method has its own limitations, therefore in our study we aimed to use several independent approaches to study the targeting of peroxisomal TA proteins. The conclusions in our manuscript are based on the results obtained from multiple methods.

5. In figure 2e, what does ‘other’ stand for? Which organelle(s) were considered for YgiM(TA) colocalization in ‘other’ category?

For the quantification of EM images, the gold particles were counted and assigned to mitochondria or peroxisomes in case the label was associated with them. If no association with mitochondria or peroxisomes was observed, the gold particle label was assigned to the 'other' category. Nucleus was excluded from this analysis.

Therefore, the 'other' category includes all other organelles (except mitochondria and peroxisomes) and cytosol. The 'other' category includes e.g. the ER and Golgi, however, the majority of the labels were located in the cytosol. For 'other' category the relative labelling indexes were <1 or ~1, which is the distribution that is expected if there is random or non-specific labelling. For the comparison, the relative labeling index for mitochondria was > 2.5 in WT, PEX3 KO or PEX19 KO cells, and for peroxisomes in WT cells it was around 170, identifying the compartments that are preferentially labeled.

Now more information on the analysis is included in the materials and methods section as well as in the figure legend for Fig 2E.

6. In figure 3, the authors showed that an endogenous peroxisomal TA protein ACBD5 is mostly localized in the mitochondria but not in the ER in PEX19 KO cell lines. Moreover, ACBD5 was barely detectable in PEX3 KO cells. The overall expression of the ACBD5 protein in PEX19 KO cell lines is very poor (figure 3e), which makes the result less convincing. One can argue the colocalization with ER is undetectable because of the expression of the ACBD5 protein in both cell lines.

The knockout of PEX3 or PEX19 disrupts peroxisomal biogenesis, which influences the synthesis and localization of peroxisomal proteins, including ACBD5 (PMID: 25177298). Thus, the expression of ACBD5 in cells lacking peroxisomes is inherently low. However, in PEX19 KO cells the ACBD5 protein level was detectable by both immunoblotting and by confocal microscopy. If ACBD5 was localized to ER at least at the same level as it was localized to mitochondria, the colocalization analysis would show this. However, we did not observe any changes in ACBD5 localization to ER upon PEX19 KO cells.

The ACBD5 protein level in PEX3 KO cells is almost completely depleted, therefore we do not make conclusions on its localization.

7. In figure 4, the authors beautifully describe the BirA/AviTag labeling approach. However, in figure 4e, why the biotin/streptavidin labeling efficiency for YgiM(TA)-AviTag protein is more efficient in the IMS fraction in comparison to the PO fraction, where the cell line Flp-In-T-Rex^TM^ HEK293T is a wild-type cell line. One would expect efficient labeling of YgiM(TA)-AviTag protein in the PO fraction and a less-efficient labeling in the IMS fraction. The authors need to comment on that.

This is an interesting observation. Indeed, the parental cells for each cell line expressing BirA construct were Flp-In T-Rex HEK293T. However, introducing BirA constructs to the different cellular compartments has different effects. Thus, we noticed that the cells expressing BirA in peroxisomes are slightly different in proliferation rate compared to the cells expressing BirA in ER or IMS. This could lead to differences in the efficiency of transfection and expression of the YgiM(TA)-AviTag construct in the particular cell line.

Another potential reason for having more labeling of YgiM(TA)-AviTag in IMS-BirA cells than in PO-BirA cells is the difference in the relative abundance of the organelles in the cells. The total mass of peroxisomes in cells is considered to be lower than the mitochondrial mass and we can clearly observe it in our EM images. This potentially leads to the more efficient labeling of the YgiM(TA)-AviTag construct in mitochondria.

Overall, there are some technical and biological limitations that do not allow our BirA/AviTag labeling approach to provide quantitative data.

8. Finally, in the discussion, the authors mentioned a possibility that the delivery of TA proteins to the peroxisomes might not be linear. It may follow a parallel pathway, which leads to a route via mitochondria. Any comment on possible mechanisms that may support this parallel pathway? Is there any information of the regulatory effect behind this linear/dual localization of TA proteins to the peroxisomes?

The targeting and delivery of tail-anchored (TA) proteins to organelles, including peroxisomes, is a complex process that may involve multiple pathways. Recent evidence (PMID: 28146471) and the data from our study suggest that both ER and mitochondria play roles in this trafficking mechanism, hinting at the possibility of parallel pathways for peroxisomal TA proteins. 

The study by Sugiura et al. (PMID: 28146471) provides evidence for the role of mitochondria in the *de novo* generation of peroxisomes in mammalian cells. This suggests that mitochondria might act as an intermediate transit or quality control point for some TA proteins, ensuring their proper folding, post-translational modifications, or even acting as a reservoir before they reach their final destination in the peroxisomes.  

The ER was implicated in the biogenesis of and communication with peroxisomes. The study by Kim et al. (2017, PMID: 28108526) elucidated the role of VAPs and ACBD5 in tethering peroxisomes to the ER, which is crucial for peroxisome maintenance and lipid homeostasis. This interaction between the ER and peroxisomes might be vital for the proper trafficking and localization of TA proteins. 

Peroxisomes, mitochondria, and ER share functional overlaps, including fatty acid metabolism and ROS handling (PMID: 26858947, PMID: 26629313, PMID: 29720625). The functional interplay might necessitate certain proteins to be present in multiple organelles, leading to dual targeting. An imbalance in ROS levels in peroxisomes can lead to alterations in mitochondrial ROS activity (PMID: 22536190). This redox interplay might influence the targeting of some proteins, suggesting a regulatory mechanism that could influence the dual localization of TA proteins. 

Furthermore, the unconventional secretion of FGF2, a protein that lacks a signal peptide, is mediated by a type I mechanism characterized by direct protein translocation across the plasma membrane. This process is regulated by interactions with specific membrane lipids and proteins, such as PI(4,5)P2 and HSPGs, and does not require ATP or a membrane potential (PMID: 28799166). It is possible that TA proteins might utilize similar mechanisms for their delivery to peroxisomes. The presence of specific lipids or proteins in the peroxisomal membrane could facilitate the direct transport of TA proteins and bypass the need for a linear pathway. Additionally, the interactions of TA proteins with other cellular components, similar to the interactions of FGF2 with ATP1A1 and Tec kinase, may play regulatory roles in modulating the efficiency of TA protein delivery to peroxisomes. The secretion pathways described for FGF2 emphasize the importance of specific lipid-protein interactions in determining the route and efficiency of protein secretion. Similar lipid-protein interactions, either at the mitochondria or ER, could influence TA protein localization and delivery to peroxisomes. For example, specific phosphoinositides or other lipids present in the mitochondrial or ER membranes might interact with TA proteins, guiding them through a parallel pathway before their final delivery to peroxisomes.

In conclusion, while the exact mechanisms and regulatory effects behind the linear or dual delivery of TA proteins to peroxisomes remain to be elucidated, insights from unconventional secretion pathways, such as that of FGF2, provide valuable perspectives on the potential complexities involved. Further studies are needed to determine the specific factors and interactions that govern the routing of TA proteins in mammalian cells.

Overall, this manuscript has potential, however, it requires further explanations of their data. Some control experiments may be required to support their findings.

Reviewer #2: The manuscript entitled “The subset of peroxisomal tail-anchored proteins do not reach peroxisomes via ER, 
instead mitochondria can be involved” where authors have demonstrated how mitochondria plays an important role for targeting peroxisomal TA proteins in both presence and absence of peroxisomes. Most importantly, authors have highlighted their findings by several approaches including immunoelectron microscopy. Moreover, authors have also discussed and are curious to investigate mechanism behind the mitochondrial targeting of Peroxisomal tail-anchored proteins.
